# Information theory and dimensionality of space

**DOI:** 10.1038/s41598-020-77855-9

**Published:** 2020-11-26

**Authors:** Subhash Kak

**Affiliations:** Oklahoma State University, Stillwater, USA

**Keywords:** Information theory and computation, Computer science

## Abstract

We present an information-theoretic approach to the optimal representation of the intrinsic dimensionality of data and show it is a noninteger. Since optimality is accepted as a physical principle, this provides a theoretical explanation for why noninteger dimensions are useful in many branches of physics, where they have been introduced based on experimental considerations. Noninteger dimensions correlate with lesser density as in the Hausdorff dimension and this can have measurable effects. We use the lower density of noninteger dimension to resolve the problem of two different values of the Hubble constant obtained using different methods.

## Introduction

Noninteger dimensions are used in various branches of physics and engineering to explain the emergence of scale invariant phenomena^1–3^. In quantum field theories, they are introduced through dimensional regularization^4–7^; since Gaussian integrals can be written as a *d*-dimensional product of a single dimensional Gaussian integral, one can generalize them to noninteger dimensions. The continuous squeezing of a three-dimensional space into a two-dimensional space also generates intermediate scenarios with noninteger dimensions^8–10^. They are introduced elsewhere to address the origins of self-similar structures in cosmology^11–14^, where the exact dimensions are chosen to fit the experimental data.

In this paper, we approach dimensionality from an information theoretic perspective related to optimal representation of data. We show that the optimal dimension associated with the representation of information is *e* = 2.71828…, a noninteger number^15,16^, and we derive it also from the perspective of the experimenter. Knowledge, as justified true belief, is normally seen as resulting from lucky guesses, but optimality provides a sounder basis to epistemology and, therefore, this can be the standard against which the inherent dimensionality of data may be compared.

In principle, the optimal dimension may be tested on data from a variety of areas. Here we consider the problem of the large-scale structure of the universe. Sky surveys and mappings of the various wavelength bands of electromagnetic radiation have yielded information on the content and character of this structure that appears to follow a hierarchical model with organization up to the scale of superclusters and filaments. A fractal dimension was computed based on the data up to a certain scale^17^. But such computations of fractal dimensions are yet not fully developed because of the incompleteness of the available data.

We apply the optimal dimensionality measure to a much simpler problem that side-steps the question of the actual dimension to be associated with the large-scale structure of the universe. We propose that physical data of expansion of the large-scale universe may be related to this optimal number, and so we should expect a discrepancy in the computations that implicitly assign the dimension of three to the data. In particular, we show that the discrepancy in the data of the Hubble constant obtained using two different methods^18–20^ can be resolved considering local- and large-scale perspectives on dimensionality of data.

## Information and dimensions

The assumption that space is three-dimensional Euclidean is the idealistic position of mathematical philosophy according to which mathematical concepts have an independent reality. But as we know it is necessary to examine all underlying assumptions in formal systems to see if they square with our intuition, which, in itself, must be tested against experimental observations. Newton in his *Principia* defined motion of material objects with respect to absolute time and space, which were left undefined. Leibniz, on the other hand, proposed a different view in which events are more fundamental than time instants. According to Leibniz, the primary role is to be ascribed to matter and its properties and it is in relation to these properties that one can speak of time and space and not in any absolute sense. Time is simply succession of instantaneous configurations of matter and not something that flows independent of the bodies in the universe.

By considering the event as primary, we can measure its information as related to its frequency: the less likely the event, the information is greater. The logarithm measure associated with information has become widely accepted because it is additive. In other words, *additivity* of information is the underlying unstated assumption in the information-theoretic approach to structure and since information is related to the experimenter, this constitutes a subject-centered approach to reality. The entropy, or the average information associated with a source, is maximized when the events have the same probability.

Although it is troublesome to visualize noninteger number of dimensions, formal analysis of such systems has been done and several studies have investigated their topological properties^21^, and also questions of differentiability^22–24^, and integration^25,26^. Noninteger dimensions provide explanation for recursion and scale-invariance in complex systems—biological, physical, or engineered—and explain fractal behavior, examples of which include the Mandelbrot set, patterns in Romanesco broccoli, snowflakes, the Nautilus shell, complex computer networks, brain structure, as well as the filament structure and distribution of matter in cosmology.

The fractal dimension is generally the same as the box-counting dimension ${dim}_{box}\left(S\right)$ of a set S in a Euclidean space ${\mathbf{R}}^{n}.$ Suppose that *N*(*ε*) is the number of boxes of side length ε required to cover the set. Then the box-counting dimension is defined as:

1 $${dim}_{box}\left(S\right)=\underset{\epsilon \to \infty }{\text{lim}}\frac{\text{ln}N(\epsilon )}{\text{ln }(\frac{1}{\varepsilon })}$$

The computed dimension is a measure of the density of the set, and to that extent it appears to embody the intuition of dimension that is based on our sense of the nature of physical space.

The algorithms to generate mathematical examples of noninteger sets incorporate self-similarity. Thus, in the well-known one-dimensional Cantor set, in which at every step we leave out the middle-third^1,2^, the total length of iteration *n* is $({2/3)}^{n}$. We need to calculate the box-counting dimension using boxes (sticks) of length ${\varepsilon }_{n}={3}^{-n}$ to cover the set of size ${N}_{box}\left({\varepsilon }_{n}\right)={2}^{n}$.

Since the iteration imply leaving out one part for every three, we get

2 $$d=\underset{\varepsilon \to 0}{\text{lim}}\left(\frac{\text{ln}{N}_{box}(\varepsilon )}{\text{ln}(1/\varepsilon )}\right)=\underset{n\to \infty }{\text{lim}}\frac{\text{ln}{2}^{n}}{\text{ln}{3}^{n}}=\frac{\text{ln}2}{\text{ln}3}\approx 0.6309$$

But note that the idea of noninteger dimensions should not be viewed only through the lens of algorithmically generated fractals and in spite of many studies on the topological properties of such spaces^21–23^, its deeper implications for understanding physical phenomena are yet to be fully investigated. In particular, one may relate the process of recursive generation of the algorithmically generated fractal to fundamental properties of space.

**Figure 1:**
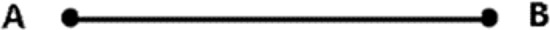
Two points separated by distance *r*.

To look at another perspective on noninteger space, assume an infinite space of dimension *d*. Let there be two objects separated by distance *r*. Building on the intuition that the density of objects in a noninteger space goes down with each iteration as in the Cantor set, the two objects of Fig. 1 would tend to come nearer, which constitutes a potential between them.

One interpretation of this *d*-dimensional space is that the objects at the two points A and B will have a potential equal to ${U}_{AB}= \frac{k(1-d)}{r}$, where *k* is a constant that depends on the nature of the two objects. When *d* = 1, that is the space is integer dimensional, there is zero potential and so there is no dynamics. The force associated with this potential will be ${F}_{AB}=\frac{dU}{dr}=\frac{-k(1-d)}{{r}^{2}}$, which constitutes an inverse-square law.

Now let us consider the question of information associated with the space. If space were *d*-dimensional, one could label the dimensions as 1, 2, 3,…*d*. The probability of the use of each of the *d* dimensions may, from considerations related to maximization of entropy, be taken to be the same and equal to $1/d$. Therefore, the information associated with each dimension is $\text{ln}d$.

Clearly, this information increases as *d* increases. But this increase must be balanced against the cost of the use of the larger dimension set. Information efficiency per dimension is:

3 $$E\left(d\right)=\frac{\text{ln}d}{d}$$

Its maximum value is obtained by taking the derivative of $E\left(d\right)$ and equating that to zero, which yields ${d}_{optimum}=e=2.71828....$ In other words:

### **Theorem**

*The optimal number of dimensions associated with representation is* *e.*

**Figure 2:**
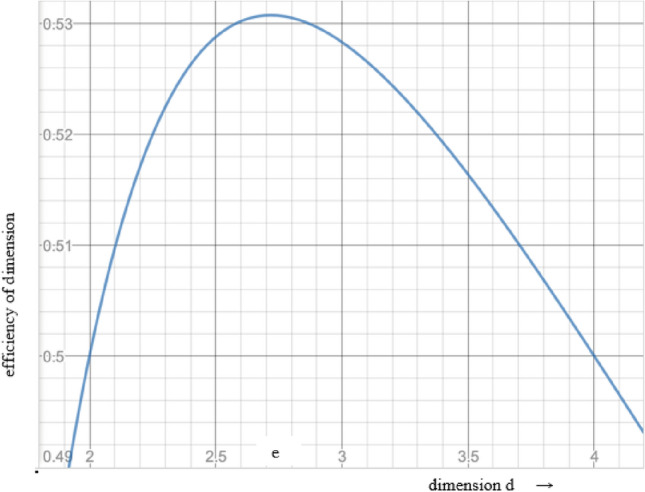
Efficiency of dimensions for *d* = 2, *e*, 3, and 4.

The optimal base value for representation of numbers is e = 2.718, and the integer closest to it, namely 3, is more efficient than binary^27,28^. The superiority of the efficiency of *e*-dimensions over two or three is evident in Fig. 2. The mathematical argument is that the *e*-dimensions are the most efficient representation of information and since Nature chooses optimality, as in physical laws such as the *Principle of Least Action*, the actual physical data should have this dimensionality.

From the perspective of visualization, the dimensionality is quite close to the fractal dimension associated with the Menger sponge^29–31^ which has a dimension of roughly 2.727. But this structure should not be viewed as anything more than a discrete analog of the physical noninteger dimensional system which is clear from the fact that different sets can have the same fractal dimension.

To determine the fractal dimension of a random self-similar system, one needs to do a computation over the actual experimental data. The Cantor set is one-dimensional data, but one can see how a similar approach leads to three-dimensional structures.

Let us now consider this problem from the perspective of the experimenter. Let the system objects be labeled 1, 2, 3, …, *n*, and the objects in the measuring instrument be labeled *n* + 1, …, *n* + *m*. The information associated with the system will be maximized when the objects are associated with equal probability; this means that the contents of the objects, which may be represented by nodes in a network, have equal significance. When the measurement nodes interact with the system, the information obtained by its nodes will be maximized when they are characterized quite the same way as the system nodes. This means that a *posteriori* probability of the nodes upon measurement goes down from 1*/n* to 1/*(n* + *m).*

The a priori information in each object is the logarithm of the inverse of its probability, or the information is $-\text{ln}\frac{1}{n}=\text{ln}n.$

The information in node *k* is $I\left(k\right)=\text{ln}n,$ and the information in the node *k* together with the measurement apparatus is $I\left(k+M\right)=\text{ln}(n+m)$. The information obtained by the observer upon measurement is $I\left(k+M\right)-I\left(k\right)=\mathit{ln}\left(n+m\right)-\mathit{ln}n=\mathit{ln}\frac{n+m}{n}$.

We can also examine it in terms of bins of potential information, and these bins are, ordinarily, the nodes on which the data resides. This data is transformed into information by virtue of the experimenter’s interaction with the physical system. We call the ratio of the system nodes and the measurement nodes as variety $V\left(k\right)=\left(\frac{n+m}{n}\right),$ which reflects the result of the exploration of one system node in terms of the dimensionality of data.

It is reasonable to assume that the novelty from each node measurement is independent and therefore the individual values should be multiplied:

$${V}_{data}={\left(\frac{n+m}{n}\right)}^{n}$$

The true variety of the data is obtained when $n\to \infty$:

4 $$V= \underset{n\to \infty }{\text{lim}}{\left(1+\frac{m}{n}\right)}^{n}={e}^{m}$$

Clearly, interrogating of the system with greater number of measurement nodes will provide increased information. An intuitively satisfactory way to define dimensionality is to compute the infimum of the *variety, V,* that can be associated with all the object (or node)-states within the system.

### Definition

The dimension,* D*, of the data is the minimum possible value of *V*.

The dimension of all linear data will be one, because such data can be placed in a single bin, and that of data associated with a plane will be two. This idea of dimension derived from information considerations appears to be consistent with its intuitive meaning.

Considering n = 1, m = 1 the space has a dimension of 2, for it is associated with the pair of states associated with the system and the observer. With n = 2, D = 2.25, that indicate correlations between the two objects and the one observer. Beyond this the value builds up to 2.718…

5 $$D= \underset{m}{\text{min}}\{{e}^{m}\}=e$$

This value of *D* may be interpreted in different ways: (i) as representing the statistical average of the fundamental relationships amongst the data, or (ii) the fundamental characteristics of the data space.

If we interpret *D* to represent the dimensions of the data space, then data obtained from astronomical problems should be associated with this dimension. In order to test this hypothesis, we consider the question of the measurement of the Hubble constant (*H*_*0*_) because it deals with the physical distribution of objects in the universe.

## Two different estimates of the Hubble constant

There are two conflicting values of the Hubble constant, based on whether one analyzes the cosmic microwave background (CMB) (the “early” universe estimate) or observes motions of stars and galaxies (the “late” universe estimate). According to the Planck Collaboration^18^ exploring the early universe, *H*_0_ is about 67 km s^−1^ Mpc^−1^, whereas a late universe estimate, Supernova H_0_ for the Equation of State (SHoES)^19^, has it at about 74 km s^−1^ Mpc^−1^.

A succession of distance indicators, which constitutes the distance ladder, is used for determining distances to other stars and galaxies. Because the more distant steps of the cosmic distance ladder depend upon the nearer ones, they include the effects of both systematic and statistical errors in the nearer steps, and due to these propagating errors, the precision is necessarily poorer for the more distant objects. Fortunately, the significance of location errors is lessened in computation of rates of change as in the case of the Hubble constant.

Planck’s estimate of *H*_*0*_ relies on measuring features in the CMB using Lambda-CDM, the standard model of cosmology. On the other hand, SHoES estimates the universe’s expansion rate by measuring distances to other galaxies from the supernovas’ distance and redshift, where brightness is related to luminosity by calibration against standard candles, which for SHoES are the Cepheid variables.

The independent Megamaser Cosmology Project^20^ considers galaxies with disks of water-bearing molecular gas orbiting supermassive black holes at galaxy centers. Considering distance measurements to four galaxies, and combined with previous distance measurements of two other galaxies, the calculations produce a value for *H*_*0*_ of 73.9 km s^−1^ Mpc^−1^ that is nearly identical to the SHoES estimate. The discrepancy between the early and late universe estimates of the Hubble constant has not yielded to any analysis and it has been termed a crisis in physics^32^.

We now present a resolution to the problem of the diverging estimates of the Hubble constant H_0_, based on early- or late- universe models. The discrepancy is between the values of 67 km s^−1^ Mpc^−1^ from the early universe and 74 km s^−1^ Mpc^−1^ from the late universe. This resolution comes from our information-theoretic perspective that implies that the optimal dimension to be associated with physical space is *e* = 2.71828. If physical reality is *e*-dimensional and we insist on viewing it as being 3-dimensional then there is a discrepancy equal to $\frac{e}{3}=0.9060.$ This number is very close to the divergence of $\frac{67}{74}=0.9054$ from the experimental data.

The efficiency of the three-dimensional space is off from the *e*-dimensional optimal space by about 0.003, or about 0.6 percent^16^. One may speculate that since our cognitions are based on counting, we associate the nearest integer space of 3 dimensions to space which leads to our normal sense about its nature.

## Conclusions

The paper presented arguments on the relationship between geometry of numbers and corresponding dimensionality. Using information theory, we showed that data from physical space has an optimal dimensionality of *e* $\approx$ 2.71828; this was also considered from the perspective of the experimenter and we obtained the same figure when the size of the data set became boundless. This provides the mathematical basis for many instances of noninteger dimensionality that are encountered in physical and engineered systems.

As illustration of this theory, we applied this idea to the question of expansion rate of the universe. Specifically, we considered the problem of the discrepancy of the Hubble constant based on early and late universe models and showed that this discrepancy disappears if one recognizes that the early universe model provides the true *e*-dimensional estimate whereas the late universe model imposes a 3-dimensional gloss on our measurements, which arises from the mathematical models that are fitted into the actual measurements.
